# Chrysin ameliorates synovitis and fibrosis of osteoarthritic fibroblast-like synoviocytes in rats through PERK/TXNIP/NLRP3 signaling

**DOI:** 10.3389/fphar.2023.1170243

**Published:** 2023-03-20

**Authors:** Liang Ding, Taiyang Liao, Nan Yang, Yibao Wei, Runlin Xing, Peng Wu, Xiaochen Li, Jun Mao, Peimin Wang

**Affiliations:** ^1^ Department of Orthopedics, Affiliated Hospital of Nanjing University of Chinese Medicine, Nanjing, Liaoning, China; ^2^ Jiangsu Province Hospital of Chinese Medicine, Nanjing, Jiangsu, China; ^3^ Key Laboratory for Metabolic Diseases in Chinese Medicine, First College of Clinical Medicine, Nanjing University of Chinese Medicine, Nanjing, China

**Keywords:** knee osteoarthritis, chrysin, synovitis, fibrosis, PERK/TXNIP/NLRP3, synovial fibroblasts

## Abstract

**Objective:** Synovitis and fibrosis are common pathological features of knee osteoarthritis (KOA). The interaction of synovitis and fibrosis can promote KOA progression. Chrysin (CHR), a natural flavonoid, may treat inflammation and prevent fibrosis. However, the effect and mechanism of CHR in KOA synovitis and fibrosis remains unclear.

**Methods:** The KOA model was established in male SD rats by anterior cruciate ligament transection (ACLT), and histological analysis was used to evaluate synovitis and fibrosis. IL-6, IL-1β and TNF-α mRNA expression in synovial tissue was measured by qRT‒PCR. Immunohistochemistry (IHC) was performed to detect GRP78, ATF-6 and TXNIP expression in vivo. Synovial fibroblasts (SFs) were treated with TGF-β1 to stimulate the inflammatory response and fibrosis. CCK-8 assays were used to detect the viability of CHR-treated SFs. The IL-1β level was detected by immunofluorescence analysis. Coimmunoprecipitation (Co-IP) and double immunofluorescence colocalization were used to detect the physiological interaction between TXNIP and NLRP3. The expression of fibrosis-related mediators and PERK/TXNIP/NLRP3 signaling molecules was detected by western blotting and qRT-PCR.

**Results:** Four weeks after CHR treatment, pathological sections and associated scores showed that CHR improved synovitis and fibrosis in the ACLT model. In vitro, CHR attenuated the TGF-β1-induced inflammatory response and fibrosis in SFs. Moreover, CHR suppressed the expression of synovial fibrosis markers and PERK/TXNIP/NLRP3 signaling molecules in the synovial tissue of rats with ACLT and cultured SFs. More importantly, we found that CHR inhibited TXNIP-NLRP3 interactions in TGF-β-induced SFs.

**Conclusion:** Our findings indicate that CHR can ameliorate synovitis and fibrosis in KOA. The underlying mechanism may be related to the PERK/TXNIP/NLRP3 signaling pathway.

## 1 Introduction

Knee osteoarthritis (KOA) is a chronic low-grade inflammatory disease of the synovium that is characterized by synovial hyperplasia, cell infiltration, neovascularization, inflammation and fibrosis (Nanus et al., 2021). Synovial fibrosis in KOA is thought to represent a terminal stage of synovial inflammation (Maglaviceanu et al., 2021). When synovitis develops improperly, fibrotic stroma accumulate and begin the tissue repair process (Maglaviceanu et al., 2021). This inferior tissue repair capacity leads to a crisis so severe that KOA patients suffer joint pain, stiffness, and disability (Wu et al., 2022). Clinically, more than 50% of OA patients are thought to have synovial fibrosis (Remst et al., 2015). Extensive research has revealed that synovial fibroblasts (SFs) are the predominant effector cells in synovitis and fibrosis (Zhang et al., 2019a). Therefore, focusing on SFs may result in the creation of cutting-edge treatments for synovitis and synovial fibrosis that could enhance the quality of life for KOA patients.

Synovitis is responsible for the production of proinflammatory cytokines such as interleukin-6 (IL-6), tumor necrosis factor-α (TNF-α), and interleukin-1β (IL-1β) (Dwivedi et al., 2022; Yoshida et al., 2022). A small amount of synovial fibrosis in the early stages fuels the regression of inflammation, and if sustained development occurs, it will inevitably develop into an end-stage lesion in the KOA synovium (Maglaviceanu et al., 2021). Synovial fibrosis has been defined as the long-term overproduction of extracellular matrix (ECM), including collagen type 1 α1 chain (COL1A1), procollagenlysine, procollagen-lysine, 2-oxoglutarate 5-dioxygenase 2 (PLOD2), tissue inhibitor of metalloproteinase 1 (TIMP1), fibronectin, and thrombospondin (Zhang et al., 2019b; Vaamonde-Garcia et al., 2019). These fibrotic markers are essential for maintaining the homeostasis of the ECM. Additionally, there is widespread knowledge that transforming growth factor-β (TGF-β) plays a role in collagen deposition during fibrosis (Ciregia et al., 2021). As a result, antifibrotic therapy with the goal of preventing fibrosis progression would be extremely beneficial for KOA.

Targeting overactive canonical PERK/TXNIP/NLRP3 signaling has emerged as a promising treatment strategy for KOA (Liu et al., 2021; Tian et al., 2023). In addition, based on the limitations of previous studies, we are also in search of drug monomers that can suppress the NLRP3 inflammasome to mitigate synovitis and fibrosis in KOA (Zhang et al., 2019b). Chrysin (CHR) is a flavonoid that has been shown to reduce chondrocyte apoptosis and treat KOA by inhibiting nuclear factor kappa-B (NF-κB) activation and the high mobility group protein 1 (HMGB1) signaling pathway (Zheng et al., 2017; Zhang et al., 2019c). Another study demonstrated that CHR relieves synovitis and pain in KOA rats by suppressing the NLRP3 inflammasome cascade (Liao et al., 2020). However, the protective effect of CHR on TGF-β-induced synovial fibers and the signaling pathway associated with PERK/TXNIP/NLRP3 is still unclear.

Here, to study the antifibrotic and anti-inflammatory properties of CHR during KOA, we used a surgically invasive model of dissociation of the anterior cruciate ligament (ACL). This model can lead to joint overload and instability that mimics the synovitis and fibrosis observed in KOA. Furthermore, SFs were isolated from Sprague‒Dawley (SD) rats and stimulated with TGF-β1 to establish a KOA cell model and investigate whether CHR can alleviate synovitis and fibrosis, as well as how PERK/TXNIP/NLRP3 signaling plays a role in this process.

## 2 Material and methods

### 2.1 Animals

SD rats were purchased from the Experimental Animal Center of Nanjing University of Chinese Medicine (Nanjing, China). First, 40 healthy adult rats weighing 180–200 g were acclimated for 7 days in the ventilated cages in the barrier facility. Prior to anterior cruciate ligament transection (ACLT) surgery, the rats were anesthetized with 3% pentobarbital sodium (100 mg/kg). A longitudinal incision was made to expose the knee joint, and the ACL was dissociated with a scalpel after patellar dislocation. The wound was then closed layer by layer with 5–0 silk. In the sham group, only the skin was incised. Following ACL rupture, the CHR-L and CHR-H groups received low-dose (10 mg/kg/d) or high-dose (25 mg/kg/d) CHR (Absin Shanghai, China) intragastrically for 28 days (Chen et al., 2021). An equal volume of vehicle was administered to KOA and sham-operated rats. The rats were euthanized after 28 days of CHR administration. The synovium of each rat was removed. A piece of the synovium was used to prepare protein and mRNA extracts, while a different portion was preserved in 4% paraformaldehyde (PFA) for histological and immunohistochemical examinations. The protocol for animal care and use was approved by the Animal Care and Use Committee of Nanjing University of Chinese Medicine (Approval number: ACU211204) and followed the National Institutes of Health Guide for the Care and Use of Laboratory Animals (NIH Publications No. 8023, revised 1978).

### 2.2 Histological analysis

Synovial tissues were fixed in 10% neutral formalin, embedded in paraffin, deparaffinized, hydrated with gradient alcohol, and then transversely sectioned for routine H&E staining. Krenn’s synovitis scoring system (grades 0–9) was used to quantify pathological changes in the synovium by observers who were blinded to the experimental groups (Krenn et al., 2006). The sum of the synovitis score was as follows: 0–1, no synovitis; two to four, low-grade synovitis; and five to nine, high-grade synovitis.

Sirius red and Masson staining were carried out according to the instructions of the kit (Solarbio, Beijing, China). Approximately 5 μm-thick synovial tissue sections were observed under a Leica DMI-3000B microscope (Leica, Germany). The degree of synovial fibrosis was evaluated by calculating the synovial fibrosis score and percentage of collagen I-positive areas with ImageJ (Version 1.74; National Institutes of Health, United States, available at http://rsbweb.nih.gov/ij/). The synovial fibrosis scoring system was as follows: absent, 0; mild, one; and diffuse, 2 (Joronen et al., 2004; Liao et al., 2021).

### 2.3 Immunohistochemistry

On the 28th day after ACLT, the rats were euthanized with an overdose of anesthetic. Synovial tissues were fixed with 10% neutral-buffered formalin and embedded in paraffin. After being decalcified, the samples were dehydrated with gradient alcohol and sectioned into 5 µm-thick slices. The sagittal sections were baked at 60°C for 2 h, deparaffinized and hydrated with gradient ethanol, washed with D-Hanks three times, permeabilized for 20 min, washed with D-Hanks three times, subjected to antigen repair for 15 min, washed with D-Hanks three times, blocked with 3% hydrogen peroxide for 15 min, rinsed with D-Hanks three times and blocked with serum at room temperature for 1 h. Then, primary antibodies {[anti-GRP78 (1:300, 11587-1-AP, Proteintech Group, Rosemont, IL, United States of America), anti-ATF-6 (1:200, DF6009, Affinity Biosciences, Cincinnati, OH, United States), and anti-TXNIP (1:200, ab210826, Abcam, Cambridge, UK)]} were added and incubated in cold storage overnight. The next day, after being incubated with HRP-conjugated IgG secondary antibodies (1:1,000 dilution) for 30 min at 37°C, the sections were rinsed with water three times, the color was developed with 3,3′-diaminobenzidine (DAB), and the sections were stained with hematoxylin, dehydrated with gradient alcohol and mounted with neutral gum. Six sections per rat were evaluated by two blinded observers, and ImageJ software was used to calculate the percentage of the positive areas for semiquantitative analysis.

### 2.4 Western blotting

An appropriate amount of synovial tissue was lysed in radioimmunoprecipitation assay (RIPA) buffer (10×volume, UU-Bio Technology Co. Ltd., Suzhou, China) containing protease and phosphatase inhibitors (1:100; New Cell & Molecular Biotech, Suzhou, China), homogenized, lysed on ice for 30 min, and centrifuged at 4°C and 12,000 rpm for 15 min. The supernatant was collected, and the protein concentrations were measured by using a BCA-500T Protein Quantification Kit (Yeasen, Shanghai, China). Aliquots of protein lysates were loaded onto a sodium dodecyl sulfate-polyacrylamide gel, transferred from the gel onto 0.22 μm polyvinylidene difluoride (PVDF) membranes (Millipore, Billerica, MA, United States), and blocked with serum for 1 h. Afterward, the membranes were incubated with the following primary antibodies overnight at 4°C: anti-TGF-β (1:1,000, BF8012, Affinity Biosciences), anti-COL1A1 (1:5,000, 67288-1-Ig, Proteintech Group), anti-PLOD2 (1:1,000, ab90088, Abcam), anti-TIMP1 (1:1,000, AF7007, Affinity Biosciences), anti-PERK (1:1,000, AF5304, Affinity Biosciences), anti-p-PERK (1:2000, DF7576, Affinity Biosciences), anti-IRE1α (1:2000, DF7709, Affinity Biosciences), anti-p-IRE1α (1:1,000, AF7150, Affinity Biosciences), anti-CHOP (1:1,000, AF6277, Affinity Biosciences), anti-TXNIP (1:1,000, ab210826, Abcam), anti-NLRP3 (ET1610-93, 1:500, HUABIO) or anti-β-actin (1:5,000, AF7018, Affinity Biosciences). The membranes were washed with TBST three times (10 min each), and the HRP-conjugated secondary antibody was added and incubated for 1 h. The protein bands were visualized with Chemiluminescence ECLPlus reagents (Biosharp, Hefei, China), and the control protein was used to normalize the gray values of the target proteins by Image Lab 6.1 software (Bio-Rad Laboratories, Hercules, CA).

### 2.5 Primary SF isolation and culture

Primary SFs were isolated from male SD rats at 4–8 weeks of age. SFs were derived from the knee synovium based on our previously reported methods (Liao et al., 2020). Briefly, the synovium was removed from the anterior, medial, and lateral compartments. The synovial tissue was digested for up to 4 h in a temperature-controlled incubator and rocked every hour in 9.5 mL of digestion solution (DMEM with 1 mg/mL collagenase type I (17100017, Thermo, Waltham, United States) and 1 mg/mL dispase (S25046-1g, Yuanye, Shanghai, China). After filtration, centrifugation, precipitation, and resuspension, knee-derived SFs were cultured in DMEM (C11995500BT, Gibco Life Technologies, United States) containing 10% FBS (41030ES76, Yeasen, China) and maintained at 37°C under 5% CO_2_. To prevent the degradation of the cell phenotype, we chose SFs within the fourth to sixth generations for subsequent experiments. SFs were serum-starved overnight before treatment and were then treated without serum. The SF processing protocol was as follows:

1 Control group: SFs were not treated except for medium replacement.
2 KOA group: SFs were exposed to 2.5 ng/mL TGF-β1 (HY-P70648, MedChemExpress, Monmouth Junction, NJ, United States) for 72 h.
3 CHR-L group: 1 μM CHR plus 2.5 ng/mL TGF-β1 for 72 h in SFs.
4 CHR-H group: 5 μM CHR plus 2.5 ng/mL TGF-β1 for 72 h in SFs.

The dose of TGF-β1 was derived from Wei et al. (Wei et al., 2021), and the dose of CHR was set according to the results of the Cell Counting Kit 8 (CCK-8) assay.

### 2.6 Cell viability assay

Cell viability was determined by the CCK-8 method according to the manufacturer’s protocol. Briefly, SFs were transferred to 96-well plates (8,000/well) for 24 h and then incubated with various concentrations of CHR (1, 5, 10, 20 and 30 μM) or DMSO alone for 24 h. Afterward, 10 μL of CCK8 was added to each well and incubated at 37°C for 2 h. Finally, a microplate spectrophotometer (Envision, Perkin Elmer, Waltham, MA) was used to measure the OD values at 450 nm.

### 2.7 Immunofluorescence analysis and confocal microscopy

Fluorescence staining was performed as described previously (Zhang et al., 2019a). Briefly, SFs were seeded on glass slides, fixed with 4% PFA for 30  min, and then permeabilized with 0.2% Triton X-100 for 15 min. Then, goat serum was used to block non-specific antigens on each slide. D-Hanks was used to rinse the samples, which were incubated with primary antibodies against TXNIP (1:250, ab210826, Abcam), anti-NLRP3 (ET1610-93, 1:250, HUABIO) or IL-1β (1:100, A10609, ABclonal) in cold storage overnight. The glass plates were rinsed the next day and then incubated with CoraLite594-or Fluor488-conjugated secondary antibodies (1:250) at room temperature in the dark, followed by labeling with Antifade Mounting Medium with DAPI for 10 min. The images were observed and captured with an LSM700 laser confocal microscope (Zeiss, Germany). The average fluorescence intensity of the cells was determined using Image-Pro Plus software (Media Cybernetics Inc.).

### 2.8 Quantitative real-time PCR

According to the manufacturer’s protocol, total RNA was extracted from synovial tissues and SFs using TRIzol reagent (Vazyme, Nanjing, China). Then, the researchers estimated the contamination by the A260/280 (between 1.8 and 2.0) and the purity and integrity by electrophoresis to confirm the quality of the RNA. Next, reverse transcription was performed by using 1 μg RNA and a PrimeScript RT Reagent Kit with gDNA Eraser (Yeasen). The reactions were performed by using SYBR qPCR Master Mix (Yeasen) on an ABI 7500 PCR system (Applied Biosystems, Life Technologies, United States). The gene expression level was normalized to endogenous GAPDH mRNA and calculated by the 2^−ΔΔCT^ method. The primer sequences are listed in Table 1.

**TABLE 1:** Sequences of primers.

Gene	Forward primer (5′–3′)	Reverse primer (5′–3′)
IL-6	CCTTCTTGGGACTGATGT	ACTGGTCTGTTGTGGGTG
IL-8	ATGGGTTTGCTAGAATGT	GTGAGGTAAGATGGTGGC
IL-18	ATC​AGA​CCA​CTT​TGG​CAG​AC	TAGGGTCACAGCCAGTCC
TGF-β	TTA​CCT​TGG​TAA​CCG​GCT​G	CTG​TAT​TCC​GTC​TCC​TTG​GT
COL1A1	CAA​GAA​GAC​ATC​CCT​GAA​GTC	GCA​TAC​ATC​AGG​TTT​CCA​CG
PLOD2	AAA​TAT​AGT​CGA​GCA​GCC​CT	TCT​TCT​ACC​AAC​TCG​TCA​CAG
TIMP1	TGA​TAG​CTT​CCA​GTA​AAG​CC	CCC​TTA​TAA​CCA​GGT​CCG​A
CHOP	TAT​GAG​GAT​CTG​CAG​GAG​G	TGA​TTC​TTC​CTC​TTC​GTT​TCC
TXNIP	TCC​GAG​TGC​AGA​AGA​TCA​G	CAC​TAA​CAT​AGA​TCA​GCA​AGG​AG
NLRP3	GAG​CTG​GAC​CTC​AGT​GAC​AAT​GC	ACC​AAT​GCG​AGA​TCC​TGA​CAA​CAC
GAPDH	GGCTCTCTGCTCCTCCC	CCGTTCACACCGACCTT

### 2.9 Coimmunoprecipitation (Co-IP)

The interaction of TXNIP with NLRP3 was detected using a Co-IP Kit (P2179S, Beyotime, China). SFs were lysed in precooled lysis buffer with a protease inhibitor cocktail (1 mL/10^7^ cells) and incubated with antibodies against TXNIP and NLRP3 at 4°C overnight. Then, protein A Sepharose was added and incubated at 4°C for 1 h. The immunoprecipitates were washed three times with wash buffer, and western blotting was used to analyze the Co-IP results.

### 2.10 Statistical analysis

Statistical analyses were performed using GraphPad Prism (Version 9.0; San Diego, CA, United States). Comparisons between the sham (control), KOA and CHR groups were performed using one-way analysis of variance (ANOVA) with Tukey’s *post hoc* analysis. The experimental results are presented as the mean ± standard deviation. A value of *p* < 0.05 was considered statistically significant.

## 3 Results

### 3.1 CHR inhibits the progression of synovial inflammation in an ACLT-induced KOA model

**FIGURE 1:**
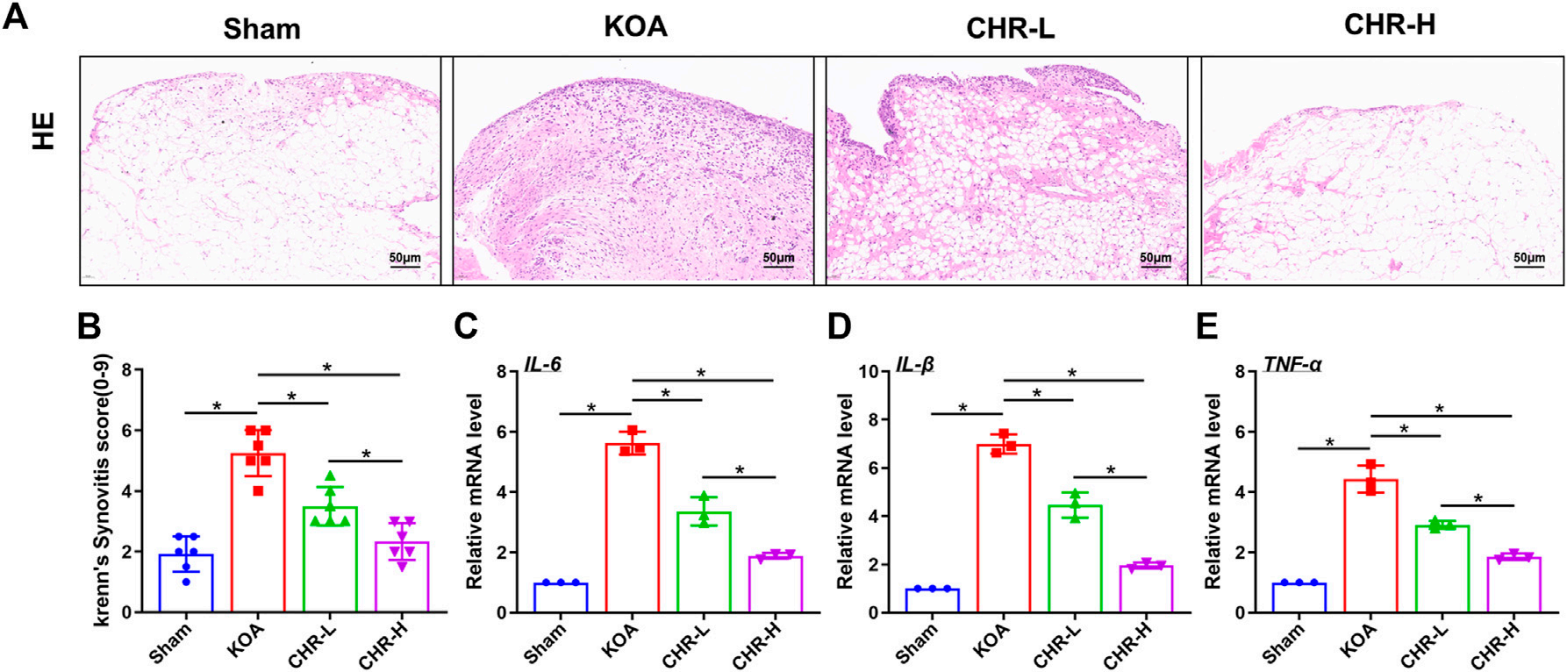
CHR reduces synovial inflammation in KOA rats. **(A)** Representative images showing H&E staining of the synovium at ×200 magnification. n = 6. Scale bars: 50 μm **(B)** Quantitative analysis of the synovitis score. **(C–E)** Quantitative PCR analysis of IL-6, IL-1β and TNF-α in synovial tissue in each group. n = 3 per group. The data in this figure are presented as the mean ± standard deviation, and statistical significance was determined with one-way ANOVA followed by Tukey’s *post hoc* analysis. ∗*p* < 0.05.

Synovial inflammation is one of the most common pathological changes associated with KOA. To observe the effect of CHR on ACLT-induced synovial inflammation in KOA, H&E staining was performed to explore characteristic changes in each group (Figure 1A). We used the classic Krenn’s synovitis scoring system, which splits the synovium into three sections for evaluation, including resident cells, the lining cell layer, and inflammatory infiltrates (Krenn et al., 2006). Each section can be scored up to three points. H&E staining revealed that CHR resulted in less resident cell hyperplasia, less formation of lining cell layers, and less inflammatory infiltration than those in the KOA group, and the Krenn’s synovitis score was significantly decreased compared with that in the KOA group (*p* < 0.05, (Figure 1B). The qRT‒PCR results demonstrated that IL-6, IL-1β and TNF-α mRNA expression in the CHR groups was significantly reduced compared with that in the KOA group (*p* < 0.05, Figures 1C–E). Taken together, these findings suggest that CHR can downregulate the expression of inflammatory mediators and alleviate synovial inflammation in KOA.

### 3.2 CHR alleviates synovial fibrosis in the ACLT-induced KOA model

**FIGURE 2:**
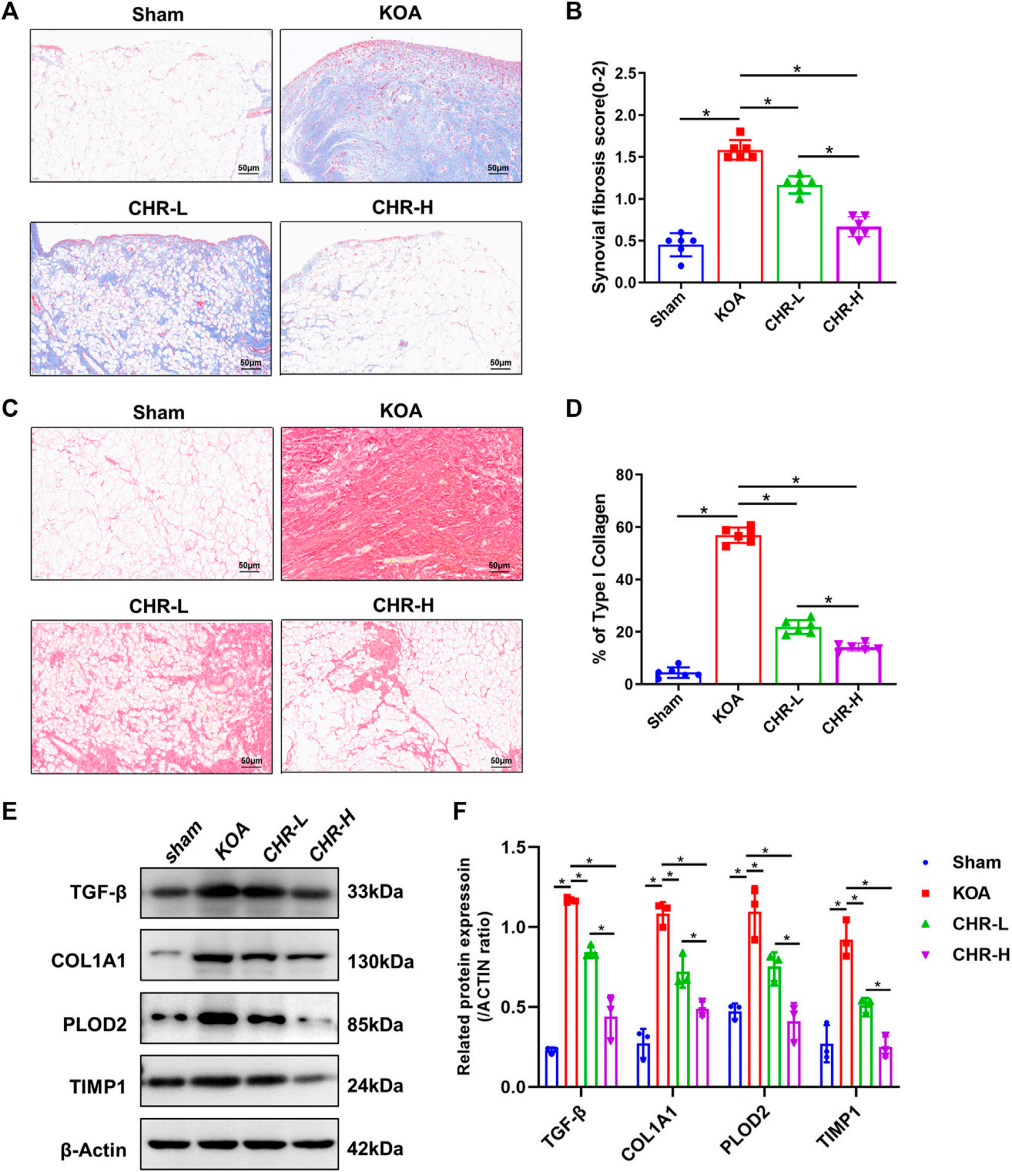
CHR alleviates synovial fibrosis in KOA rats. **(A)** Representative images of synovial sections stained with Masson and examined at ×200 magnification. n = 6. Scale bars: 50 μm. **(B)** Quantitative analysis of the synovial fibrosis score. **(C)** Representative images of synovial sections stained with Sirius red and examined at ×200 magnification. n = 6. Scale bars: 50 μm. **(D)** Quantitative analysis of type I collagen (% area) in each group. **(E)** Immunoblot analysis of TGF-β, COL1A1, PLOD2, and TIMP1. **(F)** Protein levels of TGF-β, COL1A1, PLOD2, and TIMP1 in the groups. n = 3 per group. The data in this figure are presented as the mean ± standard deviation, and statistical significance was determined with one-way ANOVA followed by Tukey’s *post hoc* analysis. ∗*p* < 0.05.

A previous study comparing the severity of synovial fibrosis in different KOA models showed that compared with the DMM- and MIA-induced KOA models, the ACLT-induced KOA model exhibited the most severe synovial fibrosis on day 28 and this effect was more profound (Zhang et al., 2022a). Therefore, we used the ACLT-induced KOA model in order to better shape the synovial fibrosis process. Masson staining is often used to evaluate tissue fibrosis, and after CHR treatment, the synovial tissue showed lighter collagen staining than that in the KOA model (*p* < 0.05, Figures 2A,B). Furthermore, we performed Sirius red staining on synovial tissue, as previous studies have confirmed that high proportions of type I collagen positivity can reveal the severity of synovial fibrosis (Zhang et al., 2019b). Sirius red staining of the synovial tissue showed that the percentage of the type I collagen-positive area in the CHR-L and CHR-H groups was decreased compared with that in the KOA group (*p* < 0:05, Figures 2C,D). Consistently, the protein levels of TGF-β, COL1A1, PLOD2, and TIMP1 in the synovial tissue of KOA rats in the CHR-L and CHR-H groups were lower than those in the KOA group (*p* < 0:05, Figures 2E,F). Overall, these results demonstrate that CHR treatment can reduce synovial fibrosis in the KOA model.

### 3.3 CHR downregulates the expression of proteins related to the PERK/TXNIP/NLRP3 signaling axis in the ACLT-induced KOA model

**FIGURE 3:**
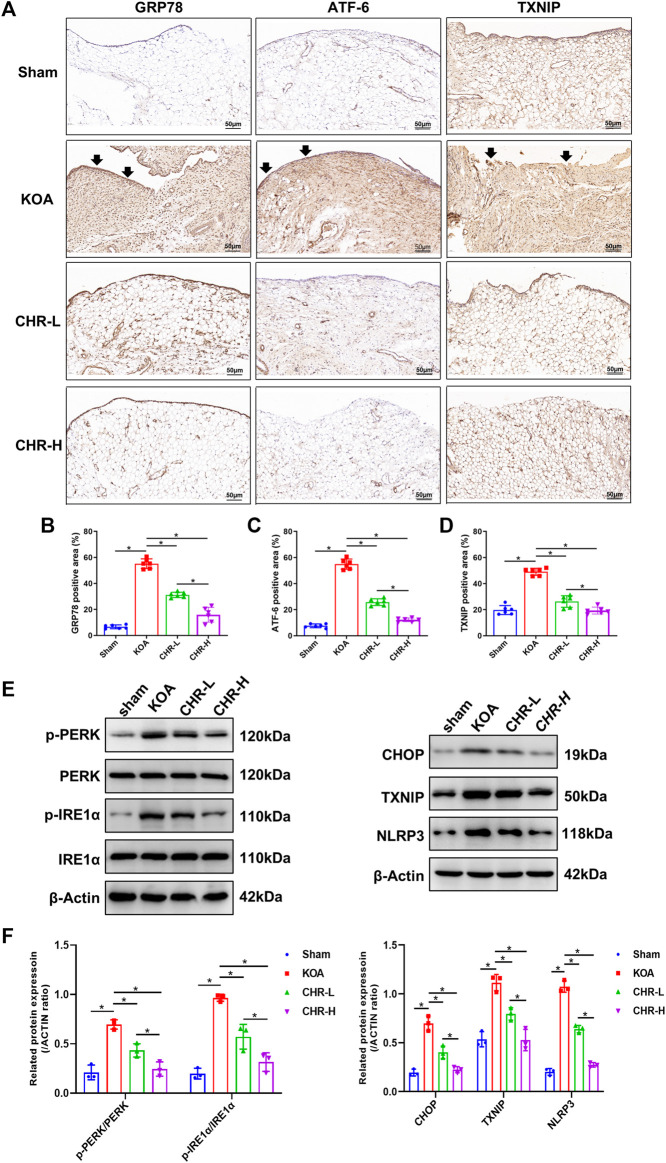
CHR downregulates the expression of proteins related to the PERK/TXNIP/NLRP3 signaling axis in KOA rats. **(A)** Immunohistochemical analysis of synovium slices from sham, KOA or CHR rats using anti-GRP78, anti-ATF-6, and anti-TXNIP antibodies at ×200 magnification. n = 6. Scale bars: 50 μm. The positive areas were mainly around the synovial membrane and vessels (black arrow). **(B–D)** The percentage of GRP78-, ATF-6- and TXNIP-positive areas in each group. **(E)** The protein levels of p-PERK/PERK, p-IRE1α/IRE1α, CHOP, TXNIP and NLRP3 were determined. **(F)** The levels of these proteins in the sham, KOA, CHR-L and CHR-H groups. n = 3 per group. The data in this figure are presented as the mean ± standard deviation, and statistical significance was determined with one-way ANOVA followed by Tukey’s *post hoc* analysis. ∗*p* < 0.05.

To determine whether CHR downregulates the expression of proteins related to the PERK/TXNIP/NLRP3 signaling axis in KOA rats, immunohistochemical analysis of synovial tissue and immunoblot analysis were performed. The percentages of GRP78-, ATF-6- and TXNIP-positive areas in the KOA group were significantly increased compared with those in the sham group (*p* < 0.05), while the CHR-L and CHR-H groups showed a significant decrease compared with the KOA group (*p* < 0.05, Figures 3A–D). The protein levels of p-PERK/PERK, p-IRE1α/IRE1α, CHOP, TXNIP and NLRP3 were measured by western blotting and were significantly higher in the KOA group than in the sham group (*p* < 0.05), while the CHR-L and CHR-H groups exhibited reduced expression compared with the KOA group (*p* < 0.05, Figures 3E,F). Collectively, these data demonstrate that CHR can reduce the expression of proteins related to the PERK/TXNIP/NLRP3 signaling axis in KOA rats.

### 3.4 CHR ameliorates TGF-β-induced synovial inflammation and fibrosis in SFs

**FIGURE 4:**
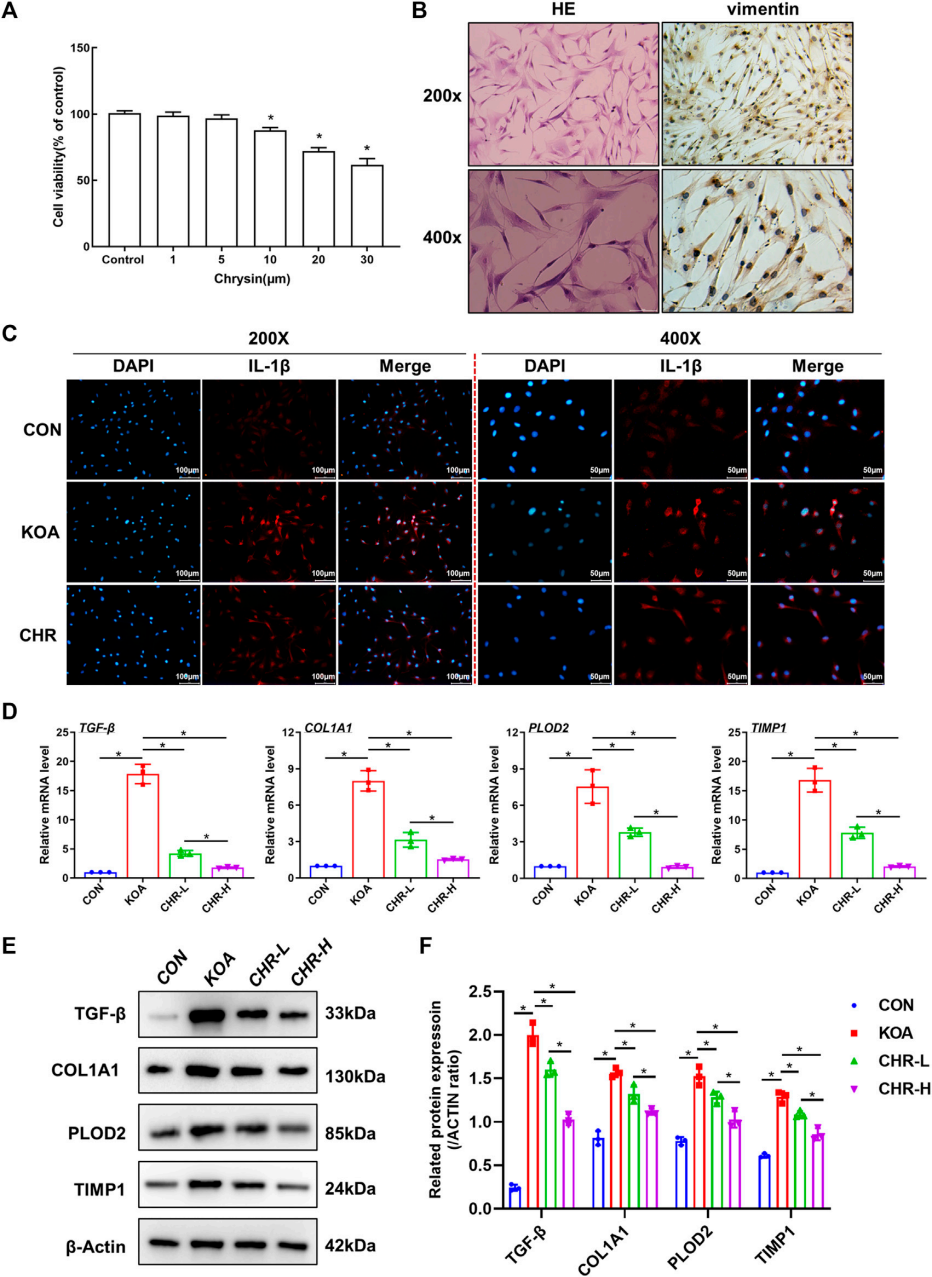
CHR ameliorates TGF-β-induced synovial inflammation and fibrosis in SFs. **(A)** HE and anti-vimentin immunostaining of SFs for cell identification. **(B)** The viability of SFs in response to CHR (1, 5, 10, 20, and 30 μM). **(C)** Representative images showing immunofluorescence analysis of IL-1β in the sham, KOA, and CHR groups at 200× or ×400 magnification. n = 3. Scale bars: 100 μm or 50 μm. **(D)** Real-time PCR analysis of TGF-β, COL1A1, PLOD2, and TIMP1. **(E)** Immunoblot analysis of TGF-β, COL1A1, PLOD2, and TIMP1. **(F)** Protein levels of TGF-β, COL1A1, PLOD2, and TIMP1 in the groups. n = 3 per group. The data in this figure are presented as the mean ± standard deviation, and statistical significance was determined with one-way ANOVA followed by Tukey’s *post hoc* analysis. ∗*p* < 0.05.

We investigated the mechanisms underlying the protective effects of CHR and proposed that CHR might protect against TGF-β-induced synovial inflammation and fibrosis. SFs were first identified by HE and anti-vimentin immunostaining (Figure 4A). CCK-8 assays were performed to investigate cell viability in response to different CHR concentrations (1, 5, 10, 20 and 30 μM) in SFs. The CCK-8 results revealed that 1 μM and 5 μM CHR were safe for SFs compared to the control group (*p* > 0.05, (Figure 4B). Therefore, 1 μM and 5 μM were chosen as the low and high doses of CHR. Numerous studies have shown the crucial role of IL-1β in synovial inflammation (Dwivedi et al., 2022; Yoshida et al., 2022). We used immunofluorescence analysis of IL-1β to investigate the anti-inflammatory effect of CHR in SFs. The results showed that CHR administration significantly decreased the level of IL-1β compared with that in the TGF-β-treated SFs (*p* < 0.05, Figure 4C). In addition, to investigate the suppression of synovial fibrosis by CHR, we performed real-time PCR and western blot analysis. As expected, the levels of TGF-β, COL1A1, PLOD2, and TIMP1 in the CHR group were significantly reduced compared with those in the KOA group (*p* < 0.05, Figures 4D–F)). Taken together, these findings suggest that CHR is a potent drug to reduce synovial inflammation and fibrosis in a TGF-β-stimulated SFs model.

### 3.5 CHR inhibits endoplasmic reticulum stress and TXNIP-NLRP3 interactions in TGF-β-induced SFs

**FIGURE 5:**
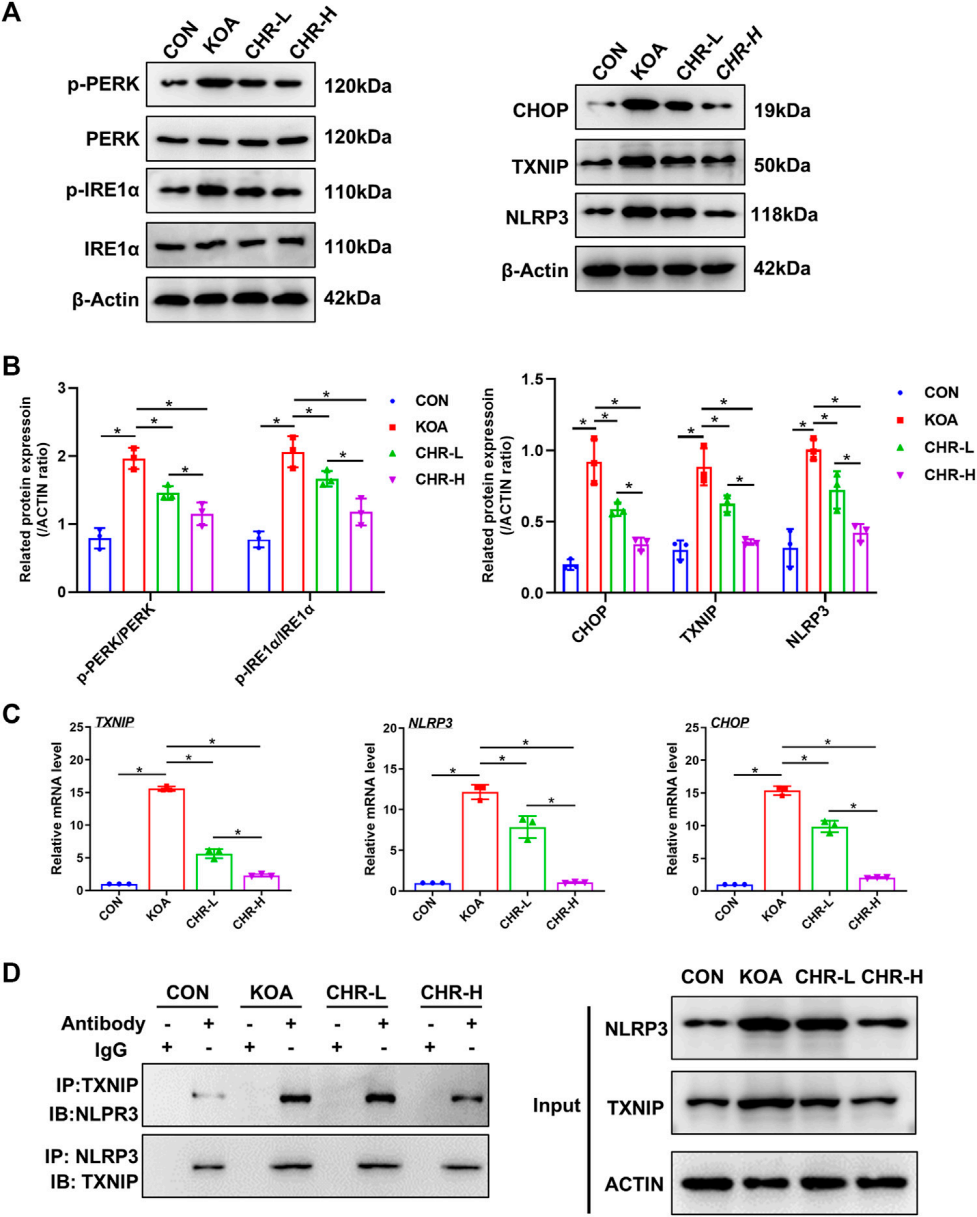
CHR inhibits endoplasmic reticulum stress and TXNIP-NLRP3 interactions in TGF-β-induced SFs. **(A)** The protein levels of p-PERK/PERK, p-IRE1α/IRE1α, CHOP, TXNIP and NLRP3 were determined. **(B)** The levels of these proteins in the CON, KOA, CHR-L and CHR-H groups. **(C)** Real-time PCR analysis of TXNIP, NLRP3 and CHOP. **(D)** Co-IP was used to detect the physiological interaction between TXNIP and NLRP3. IB: immunoblot; IP: immunoprecipitation. n = 3 per group. The data in this figure are presented as the mean ± standard deviation, and statistical significance was determined with one-way ANOVA followed by Tukey’s *post hoc* analysis. ∗*p* < 0.05.

**FIGURE 6:**
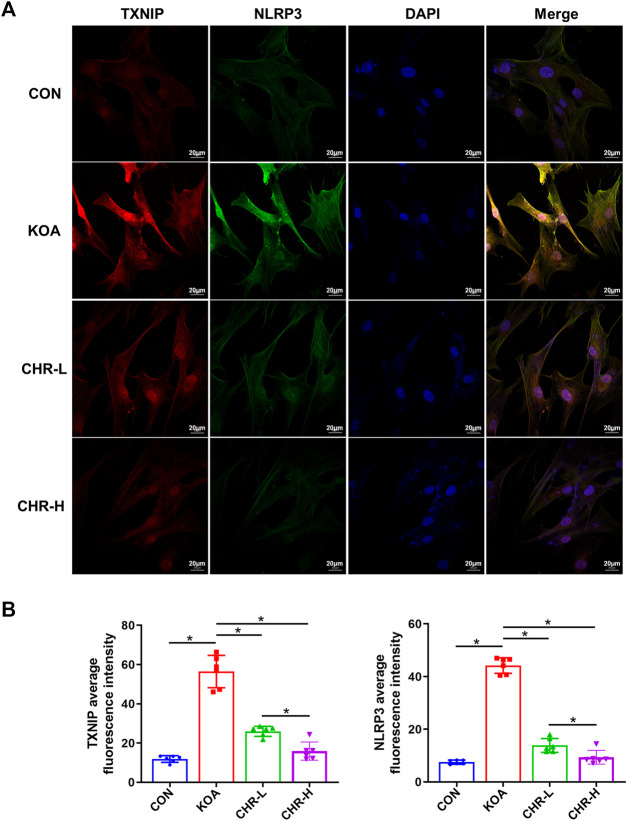
CHR prevents the colocalization of TXNIP and NLRP3 in TGF-β-induced SFs. **(A)** Confocal microscopic images of treated SFs that were immunostained with anti-TXNIP (red), anti-NLRP3 (green), and DAPI (blue). **(B)** Double immunofluorescence colocalization of TXNIP and NLRP3 at ×630 magnification. n = 6. Scale bars: 20 μm. The data in this figure are presented as the mean ± standard deviation, and statistical significance was determined with one-way ANOVA followed by Tukey’s *post hoc* analysis. ∗*p* < 0.05.

After CHR treatment for 24 h, the protein levels of p-PERK/PERK, p-IRE1α/IRE1α, CHOP, TXNIP and NLRP3 were measured by western blotting and were significantly higher in the KOA group than in the control group (*p* < 0.05), while the CHR-L and CHR-H groups exhibited reduced expression compared with the KOA group (*p* < 0.05, Figures 5A,B). The mRNA levels of CHOP, TXNIP and NLRP3 in SFs were significantly upregulated in the KOA group compared with the control group (*p* < 0.05), and the CHR-L and CHR-H groups showed downregulation compared with the KOA group (*p* < 0.05, Figure 5C). To evaluate whether NLRP3 inflammasome activation was due to an increase in TXNIP expression, Co-IP was used to confirm the hypothesis that TXNIP and the NLRP3 inflammasome could bind and interact with each other. Moreover, these interactions were reduced by CHR treatment in SFs (Figure 5D). Finally, we analyzed TXNIP and NLRP3 by performing double immunofluorescence colocalization. We found that the average fluorescence intensity of TXNIP and NLRP3 was increased in the KOA group, and CHR inhibited this increase (*p* < 0.05, Figures 6A,B). Collectively, these data demonstrate that CHR inhibits endoplasmic reticulum stress and TXNIP-NLRP3 interactions in TGF-β-induced SFs.

## 4 Discussion

KOA is a degenerative disease of the knee joint that is characterized by the progressive destruction of articular cartilage and surrounding soft tissue, especially synovial tissue, causing joint pain, swelling, stiffness, dysfunction, and even disability (Sharma, 2021). For a long time, KOA was thought to be a chronic degenerative disease caused by the wear and tear of cartilage alone. However, synovitis is now recognized as an initiating and driving factor in the development of OA (Han et al., 2020). Increasing evidence suggests that synovial inflammation occurs not only in the early stages of the disease but also multiplies with fibrosis in the late stages, resulting in clinical symptoms such as swelling and stiffness in KOA (Sunk et al., 2022). When synovitis and fibrosis occur, proinflammatory mediators (e.g., IL-6, TNF-α, IL-1β) are released into the joint space. These mediators attract immune cells and are a major cause of joint damage and pain (Liang et al., 2022). Therefore, it is important to develop reliable and effective therapies for synovitis and fibrosis.

Since the pathogenesis of KOA is poorly documented, there are no medications that can slow or reverse its progression (Oo et al., 2018). Although non-steroidal anti-inflammatory drugs (NSAIDs) are used in the clinic to relieve joint pain and swelling, they do not treat the disease completely, and excessive use can even trigger serious adverse effects in some cases. The only currently available approach for treatment of end-stage KOA is artificial joint replacement (Poulet and Staines, 2016). Therefore, a powerful and safe medication is required to treat OA. Recently, due to the lack of negative side effects, flavonoids have recently drawn the interest of researchers in the context of KOA treatment.

CHR is a flavonoid with a variety of pharmacological activities that is found in many plants, such as *Mucuna pruriens* and *Scutellaria baicalensis*, and it has anti-inflammatory, antioxidative stress, and antiapoptotic effects, as well as notable protective effects on KOA cartilage (Mani and Natesan, 2018). CHR has been shown to reduce IL-1β-induced apoptosis and inflammation in human OA chondrocytes by inhibiting activation of the NF-κB and HMGB1 signaling pathways and promoting chondrocytes to secrete type II collagen and maintain cartilage function (Zheng et al., 2017; Zhang et al., 2019c). Our previous study showed that CHR could ameliorate KOA synovial inflammation *via* the NLRP3 inflammasome, but further research is still needed (Liao et al., 2020). First, the upstream mechanisms of the NLRP3 inflammasome are unknown; second, different concentrations of CHR must be used rather than a single concentration to enhance validation; and finally, CHR only targets synovial inflammation, and synovial fibrosis has not been studied in depth.

A large number of pathological factors may contribute to fibrosis, such as TGF-β. TGF-β is the best-known profibrotic factor and is involved in fibrotic processes in almost all tissues, such as fibrotic lesions in liver, lung, kidney, skin and heart tissue (Henderson et al., 2020). To prevent fibrosis, inhibiting the expression of TGF-β would undoubtedly be a good option, as it is upstream of many cascade responses. However, TGF-β regulates several important cytokines associated with pathological activity. For instance, inhibiting TGF-β expression in synovial tissue could reduce synovial fibrosis in KOA (Remst et al., 2014). However, given that TGF-β inhibits collagen deposition and is essential for preserving the homeostasis of the ECM environment, its absence will undoubtedly make it more difficult to maintain cartilage homeostasis in the knee (Du et al., 2023). Thus, TGF-β is a double-edged sword. Therefore, focusing on downstream fibrosis-associated collagen and cross-linking enzymes that are specifically regulated by TGF-β has become crucial, and COL1A1, PLOD2 and TIMP1 are the most intensively studied (Tsezou, 2014). COL1A1 is directly involved in type I collagen synthesis and participates in a variety of pathological processes, such as tissue fibrosis, ligament injury, and cartilage destruction (Remst et al., 2014). PLOD2 is required for the stability of intermolecular cross-linking, and its overexpression increases collagen cross-linking in synovial tissue, decreases collagen turnover, and ultimately worsens fibrosis (Remst et al., 2013). TIMP1 belongs to the matrix metalloproteinase inhibitor family, is found in human and murine synovial specimens of end-stage OA and causes ECM destruction and fibrosis (Zhang et al., 2019b).

The current study demonstrates that CHR can alleviate synovitis and fibrosis in an ACLT-induced animal model and TGF-β-induced cell model. We used the ACLT surgical model because our previous studies demonstrated that this method could significantly induce synovitis and fibrosis (Zhang et al., 2022a). TGF-β1 was used at a concentration of 2.5 ng/mL because Wei et al. (Wei et al., 2021). Discovered that this concentration promoted maximum SF proliferation and upregulated fibrosis-related marker expression. In this study, we investigated whether CHR reduced synovitis and fibrosis *in vivo* and *in vitro*. For the *in vivo* part, we used H&E staining and qRT‒PCR to assess the severity of synovitis after ACLT. We also used Sirius red staining, Masson’s trichrome staining and western blotting to detect the pathological changes in synovial fibrosis. Histological staining indicated that the flavonoid CHR reduced resident cell hyperplasia, the formation of lining cell layers, inflammatory infiltration, collagen deposition and the Krenn/fibrosis score compared with those in the KOA group. The expression of fibrosis-related genes, including TGF-β, COL1A1, PLOD2 and TIMP1, was also significantly reduced. The *in vitro* experiments with SFs obtained the same results. These findings suggest that CHR can reduce ACLT- or TGF-β-induced synovitis and fibrosis.

Furthermore, prior research has demonstrated that classic PERK/TXNIP/NLRP3 signaling predominantly occurs in OA, which increases SF proliferation, increases collagen deposition and worsens synovial fibrosis (Liu et al., 2021; Tian et al., 2023). The endoplasmic reticulum stress-mediated unfolded protein response (UPR) typically involves three receptor proteins: activating transcription factor 6 (ATF-6), protein kinase RNA-like ER kinase (PERK) and inositol requiring enzyme 1α (IRE1α) (Li et al., 2020). These three receptors are inactive under physiological conditions by binding to glucose regulated protein 78/immunoglobulin binding protein (GRP78/BiP) (Huang et al., 2022). When endoplasmic reticulum stress is stimulated, GRP78 is activated and dissociates, and PERK catalyzes eukaryotic translation initiation factor 2α (eIF2-α) phosphorylation, increases CCAAT/enhancer-binding protein homologous protein (CHOP) expression, and activates the TXNIP-NLRP3 inflammatory vesicle complex, which ultimately leads to inflammation and fibrosis (Ayaub et al., 2016; Wu et al., 2018; Zhang et al., 2022b; Dong et al., 2022). In our study, we proved that CHR inhibited the expression of GRP78, ATF-6, TXNIP and the overproduction of PERK, IRE1α, CHOP, TXNIP and NLRP3 at the protein and mRNA levels. Moreover, the results showed that in rat KOA SFs, CHR significantly inhibited the expression of IL-1β induced by TGF-β, as shown by immunofluorescence analysis. Moreover, double immunofluorescence colocalization and Co-IP confirmed that TXNIP and the NLRP3 inflammasome could bind and interact with each other. Overall, our study proved the value of CHR in treating KOA synovitis and fibrosis.

## 5 Conclusion

In the current study, we sought to determine how CHR affected KOA synovitis and fibrosis in an ACLT model, as well as in cultured SFs that were isolated from the synovial tissue of SD rats, and further explored the mechanism of PERK/TXNIP/NLRP3 signaling in this process. Our results demonstrated the therapeutic effect of CHR in KOA treatment.

## Data Availability

The raw data supporting the conclusions of this article will be made available by the authors, without undue reservation.
